# Ultra-high dose rate electron FLASH beam irradiation using a modified clinical linear accelerator

**DOI:** 10.1371/journal.pone.0346577

**Published:** 2026-04-17

**Authors:** Gyu-Seok Cho, Kyo-Tae Kim, Soon-Sung Lee, Jeong-Hwan Kim, Dong-Han Lee, Hong-Suk Chang, Sang-Hyoun Choi, Kum-Bae Kim, Tae-Keun Yang, Dong-Hyun An, Geun-Beom Kim, Kun-Uk Kang, Jin-Kyu Kang, Eun-Ji Kim, Eun-Kyung Paik, Mi-Sook Kim, Won-Il Jang, Kwang-Mo Yang

**Affiliations:** 1 Radiation Therapy Technology and Standards, Korea Institute of Radiological and Medical Sciences, Seoul, Korea; 2 Department of Research and Development, GEMSS Healthcare Co., Ltd, Goyang, Korea; 3 Department of Cyberknife, Korea Institute of Radiological & Medical Sciences, Seoul, Korea; 4 Department of Radiation Oncology, Korea Institute of Radiological & Medical Sciences, Seoul, Korea; 5 Department of Radiation Oncology, Seoul Metropolitan Government - Seoul National University Boramae Medical Center, Seoul, Korea; Chung-Ang University Gwangmyeong Hospital, KOREA, REPUBLIC OF

## Abstract

FLASH radiation therapy reduced radiation-induced damage to normal tissue, compared to conventional radiation therapy, and active studies have been undertaken worldwide. While a number of preclinical studies have revealed the effectiveness of this novel radiotherapy, the biological effects have not been fully elucidated. Here, we implemented ultra-high dose-rate FLASH beam irradiation through machine modifications of the core components of an existing clinical linear accelerator, to build a database containing information related to the biological effects of the FLASH beam. We established a protocol that enables a quick conversion between the CONV and FLASH modes of an existing clinical linear accelerator. At 100 cm source-to-surface distance in 10 cm × 10 cm applicator conditions, we achieved FLASH electron beam irradiation characterized by 9.49 MeV mean energy, effective field size (95% iso-dose distribution) ∅ 39.9 mm, and maximum dose rate of 339.1 Gy/s. Irradiation of an elliptical Gaussian beam with flatness of 12.7% and symmetry of 0.3% was confirmed. Furthermore, most of the required preclinical conditions were satisfied. However, although the pulse dose value obtained from the protocol is expected to meet the requirement of 1 Gy/pulse, this result was not confirmed in this study, and further investigation is required.

## Introduction

Approximately 52% of cancer patients worldwide are being treated with radiation therapy [1]. In particular, external beam radiation therapy using Linear Accelerator (LINAC) is a treatment method that accurately targets tumor tissue through treatment planning and delivers high energy radiation. It is an effective treatment technology for inducing cell death through damage to tumor tissue [2]. As mentioned above, although this treatment method aims to accurately target the tumor mass, due to its nature, inevitable normal tissue damage induces in the process of the delivered radiation reaching the intended target [2]. The side effects caused by normal tissue damage appear differently depending on individual factors of patients, such as sex, age, immune status, and lifestyle, and has a serious impact on the patient’s quality of life in the long term. Thus, the necessity of developing a technology for its prediction and diagnosis [2–4]. Therefore, recent radiation therapy technology has focused on development a modality using a particle beam such as heavy ion and protons to minimize the damage to normal tissues while maximizing the treatment effectiveness and many application techniques such as new treatment planning techniques and beam delivery methods, are being developed [5–8].

Among these radiation therapy technologies, FLASH radiation therapy (RT) is a treatment method by delivering a ≥ 40 Gy/s ultra-high dose rate beam higher than the dose rate of conventional radiation therapy (approx. 0.01–0.4 Gy/s), which has been report to effectively improve normal tissue sparing while maintaining its tumor control ability, and it has been reported that the higher dose rate, the prominent the biological effect through various pre-clinical study worldwide [9–15]. The mechanism of the sparing effect in FLASH-RT has not yet been completely identified, but the most well-known hypothesis is the effect of rapid oxygen depletion; This is explained as mechanism in which radio-resistance increases due to a rapid drop in the oxygen partial pressure of normal tissue cells when the high dose rate radiation of 40 Gy/s or more is irradiated within the short time (≤ 0.4 s) [16–21]. For the clinical application of FLASH beam with these clinical advantages, various research groups are investing a lot of time and resources in developing equipment dedicated to FLASH beams. According to J. Bourhis et al. a research team at Lausanne University Hospital, Switzerland, the team developed Oriatron eRT6 capable of irradiating 200 Gy/s ultra-high dose rate (UHDR) beam with nominal energy 5–6 MeV electron beam and reported that it was applied to patient treatment for the first time in the World [22,23]. However, since the development of FLASH-only modality requires a lot of time and resources and a treatment planning and feedback system required for treatment, it is difficult to access for small research groups aimed at identifying mechanisms for FLASH-RT. Therefore a new research platform is being required.

In order to develop the research platform for FLASH-RT, many studies are being performed to modify an existing clinical LINAC [24–37]. Table 1 shows the summarized characteristics of FLASH electron beams irradiated by modified clinical linear accelerators.

**Table 1:** Characteristics of the FLASH electron beam using modified clinical LINAC.

Implement method	Research group	Machine	Energy [MeV]	SSD [cm]	Dose Rate [Gy/s]
Carousel, X-ray target	This Study	Varian iX	9	100	339.1
	Garty et al. [26]	Varian Clinac 2100C	9	20	600
	Szpala et al. [27]	Varian iX	17.9	80	100
	Rahman et al. [28]	Varian Clinac 2100 C/D	10	100	290
RF power, E-gun	Oh et al. [29]	Varian Clinac 23EX	16	Accessory	> 680
	Xie et al. [30]	Elekta Synergy	6	13-15	500
	Cetnar et al. [31]	Varian Clinac iX	16	100	197
RF power, E-gun, Grid	Dal Bello et al. [32]	Varian TrueBeam	16	100	256
RF power	No et al. [33]	Varian TrilogyTM	17.5	70	82.01
E-gun, Magnetron Frequency	Konradsson et al. [34]	Elekta Precise	10	100	> 200
E-gun, Bending magnet, Wedge, scattering foil	Deut et al. [35]	Elekta SL	10	100	83.6
Others	Schüler et al. [36]	Varian Clinac 21EX	20	Mirror	220
	Lempart et al. [37]	Elekta Precise	8	Wedge	300

Many previous studies have reported FLASH beam generation through manipulation of RF power and E-gun parameters. However, such approaches may present challenges for immediate clinical implementation. In this context, we explored a FLASH beam generation platform based on the exclusion of accelerator components, rather than parameter tuning, and reviewed representative studies adopting this approach; Garty et al. [26], Szpala et al. [27], and Rahman et al. [28]. The three representative studies share a common approach in achieving FLASH beam generation in X-ray mode by modifying or removing specific components, such as the carousel and X-ray target. However, each study implements this approach under different experimental configurations.

Garty et al. [26] reported FLASH beam measurements obtained within the gantry, while Szpala et al. [27] implemented beam control through console software rather than a dedicated gating system. In addition, Rahman et al. [28] achieved FLASH beam generation by adjusting the energy switch, x-ray target, and carousel with the air drive disabled. Although these approaches demonstrate the feasibility of FLASH beam generation under diverse configurations, the resulting operating conditions remain an area requiring further investigation, highlighting the need for continued development of robust and adaptable research platforms.

In this study, to develop a FLASH-RT research platform, we installed a clinical LINAC (Varian Clinac iX, Varian Medical System Inc., USA) for research only at the Korea Institute of Radiological & Medical Sciences (KIRAMS) and developed a gating system that allows control of irradiation time according to the user’s needs; the medical linear accelerator used in this study was installed at KIRAMS under regulatory approval and was dedicated exclusively to non-clinical research, with no use in patient treatment. Ultimately, this study aims to present an improved modifying protocol for clinical LINAC and report the characteristics of FLASH electron beam implemented through it.

## Experiment methods

### Machine modifications

In this study, to implement UHDR mode (denoted, FLASH mode) using Varian Clinac iX installed for research purposes, mechanical modifications were performed on several key components of the LINAC, including the carousel, X-ray target, and servo circuits. All experimental procedures were conducted in compliance with institutional radiation safety regulations, and all accelerator configurations were implemented within the operational scope defined by the manufacturer.

Previous studies have proposed several approaches to generate FLASH electron beams using modified clinical LINAC systems. Although the mechanical modification procedures, such as modifications to the carousel, X-ray target, and servo circuits, are generally similar among these studies, the methods used to control the FLASH beam differ. For example, Garty et al. [26] introduced a delay control system between the electron gun and the klystron to modify the beam timing, while Szpala et al. [27] utilized the service mode MU setting provided by the Varian system to generate FLASH beams. Rahman et al. [28] implemented a gating system combined with ionization chamber feedback to control the pulse structure.

In contrast, the present study implemented a gating system similar to that used in Rahman et al. [28]; however, the FLASH beam was controlled using a user-defined timer based on the relationship between dose rate and delivered dose in the Varian service mode. This approach enables FLASH beam generation without additional hardware modification or beamline feedback systems, providing a simpler and more practical method for implementing FLASH beams using a clinical LINAC.

The LINAC modification procedure proposed in this paper is very similar to that reported by Rahman et al. [28], but can be considered an improved approach in that the air drive remains active. In the vacuum system, mechanical adjustments of key LINAC components are pneumatically controlled by an air drive. Keeping the air drive active enables faster and more convenient mechanical adjustment of these components when switching to FLASH mode. When the air drive was turned off, the energy switch had to be mechanically restricted; however, our method allows this step to be completely omitted. Table 2 outlines the modification procedure for FLASH mode implementation.

**Table 2:** Protocol for implementing FLASH mode electron beam using the clinical LINAC.

Step	Description	LINAC parts affected
1	① Select service mode in the treatment console	Carousel
	② Remove the lead shielding from the front of the gantry head so that the carousel port.	
	③ Check the position of the open port cover in the carousel	
	④ Change the carousel position to Manual so that the electron beam can pass through the open port.	
2	⑤ Remove the lead shielding from the side of the gantry so that the target actuator is visible.	Target
	⑥ Adjust the position of the target actuator by setting the energy to Electron mode.	
	⑦ The target actuator is mechanically restricted from drifting to X-ray mode.	
3	⑧ Turn off the dose, dose rate, and beam steering servos in the electronics cabinet.	Servos Circuits
	⑨ In service mode, inter-locks for related elements are overridden.	
	⑩ Select Photon mode to deliver electron beams.	

To manually operate the energy switch and prevent the X-ray target from entering the beam path, the outer plastic accelerator cover was removed to provide physical access to the internal components. This cover does not contribute to radiation shielding and is not required for beam production; therefore, its removal did not affect beam delivery or radiation safety conditions. In addition, the carousel housing the flattening filter and scattering foil is normally obscured by front lead shielding. For the FLASH beam configuration, the carousel was manually adjusted to an open port position, enabling direct delivery of the electron beam to the phantom without attenuation by beam-modifying components.

### Electrical control circuit for gating system

A precisely designed gating system is required to deliver the electron beam within the exact irradiation time through the implemented FLASH mode. In the case of conventional mode (denoted, CONV. mode), the beam is controlled by the monitoring chamber installed in the gantry head, but in the FLASH mode, this should be inactivated because it exceeds the defined dose rate limit. To ensure consistent and reproducible FLASH beam irradiation, we developed an Opto-coupler circuit capable of delivering on/off beam signals to the gating box connected to the gating system using a micro-controller (Arduino Uno, Atmel Co., USA). Using this circuit, through the time setting of the code written using the Arduino Integrated Development Environment (IDE), the generated gating signal was transmitted to the gating switch box (Varian Medical System Inc., USA) so that the beam was irradiated for the set time.

### Machine output calibration for CONV. mode

**Fig 1:**
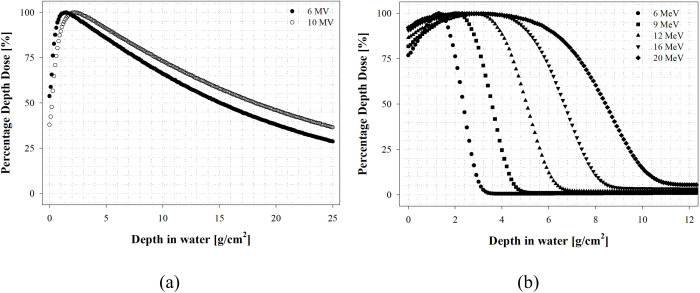
Conventional mode beam characteristics under the reference condition. **(a)** Photon beam, **(b)** Electron beam.

We performed the output calibration according to the recommendations of International Atomic Energy Agency (IAEA) TRS-398, an international protocol, to ensure the traceability of dosimetry of the CONV. mode [38]. In the case of the FLASH beam, since there is no international protocol for the reference condition, a gradual examination of traceability from the conventional dosimetry framework was necessary to minimize uncertainty in the dose. Fig 1 shows the beam characteristics for CONV mode under the reference condition.

In the case of the photon beam, a Farmer-type chamber (TM30013, PTW-Freiburg GmbH, Germany) was placed at a water depth of 10 cm under conditions of source-to-surface distance (SSD) 100 cm and a field size of 10 cm × 10 cm. An electrometer (UNIDOS Webline, PTW-Freiburg GmbH, Germany) was used to perform output calibration; 6 MV –0.03% (*TPR*_*20,10*_: 0.668), 10 MV 0.22% (*TPR*_*20,10*_: 0.738). *TPR*_*20,10*_ is defined as the photon beam quality index and refers to the ratio of dose values at 10 cm and 20 cm on the percentage depth dose (PDD). For the electron beam, the Roos Electron Chamber (TM34001, PTW-Freiburg GmbH, Germany) was placed at the reference depth (*z*_*ref*_) under conditions of SSD 100 cm and field size of 10 cm × 10 cm. The output calibration was performed at a dose rate of 400 MU/min; 6 MeV 0.41% (*R*_*50*_: 2.35 g/cm^2^), 9 MeV –0.27% (*R*_*50*_: 3.57 g/cm^2^), 12 MeV –0.01% (*R*_*50*_: 5.05 g/cm^2^), 16 MeV 0.35% (*R*_*50*_: 6.67 g/cm^2^), and 20 MeV –0.26% (*R*_*50*_: 8.43 g/cm^2^). *R*_*50*_ is defined as the electron beam quality index and refers to the depth on the PDD curve at which the dose reaches 50% of the maximum dose.

### Radiochromic film calibration

In this study, EBT-XD radiochromic film (Ashland Inc., Covington, KY) was used as a reference dosimeter to perform dosimetry for the implemented FLASH mode. In the case of the ionization chamber, which is widely used as a reference dosimeter in clinical settings, it is difficult to calibrate the dose rate dependence caused by the ion-recombination effect and the polarity effect in the FLASH mode. Furthermore, it is impossible to ensure traceability due to saturation [22]. The EBT-XD film has higher sensitivity and superior anisotropy and stability than the EBT3 radiochromic film used in clinical settings, and a limited dose rate dependence (within approximately 5%) has been reported over a wide range from CONV beam (0.078 Gy/s) to UHDR (1.5 × 10^10^ Gy/s), indicating its suitability for FLASH dosimetry [28,39,40].

**Fig 2:**
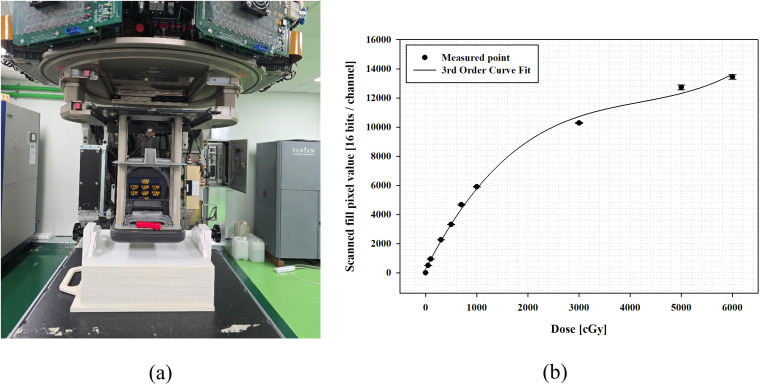
Experimental set-up for radiochromic film calibration. **(a)** Experiment set-up, **(b)** EBT-XD calibration curve at red channel.

We used a 9 MeV electron beam to obtain a calibration curve for the EBT-XD film. According to the IAEA TRS-398 protocol, plastic phantoms are recommended to be used in low-energy electron beams R_50_ ≤ 4 g/cm^2^); thus, 9 MeV electron beams, corresponding to high energy beams from the applicable energy range, were selected. The EBT-XD film was placed on *z*_*ref*_ in the SP34 slab phantom and irradiated in 10 levels in the range of 0–6000 cGy, under conditions of SSD 100 cm and field size of 10 cm × 10 cm. The SP34 slab phantom is made of RW3 materials (Polystyrene 98% + TiO_2_ 2%); *ρ*_*pl*_ 1.045 g/cm^3^, effective material parameters ((Z/A)_eff_) 0.536, and electron density is 1.012 times higher than that of water [41]. After the irradiation, the film reading was performed after 24 hours, and the flatbed scanner (Epson Expression 10000XL, EPSON America, Inc., USA) was stabilized through a warm-up time of 15 minutes and three scans on non-exposure film. Films were acquired in TIFF format with a resolution of 72 dpi and 48-bit RGB (16 bits per channel), with all image enhancement filters turned off. Background correction was performed by subtracting the pixel values of un-irradiated films from those of the irradiated films. Only the red channel was used for analysis, as it provides the highest sensitivity and most consistent response for EBT-XD films, with uncertainties within 2.3% in the 5–40 Gy dose range when calibrated at 6 and 20 MeV [42]. The net optical density values at the ten dose points were fitted using a spline model in the commercial software (DoseLab Ver. 6.80, Mobius Medical Systems, USA) to generate the final calibration curve, which was then used to convert optical density measurements to dose in all subsequent analyses. Fig 2 shows the experimental setup to obtain the calibration curve for the EBT-XD film and the calibration curve.

### FLASH dosimetry

In this study, PDD, beam profile, and beam output were evaluated to perform beam characterization of the implemented FLASH mode. We changed the photon beam mode 10X in the treatment console. In addition, in order to compare the characteristics of the FLASH mode with those of the previous studies, the experiment was also conducted under a condition without an applicator. Table 3 shows the measurement conditions for evaluating FLASH mode characteristics.

**Table 3:** Measurement condition.

Irradiation Time	50, 100, 200, 300, 400 ms
Dose rate	100, 200, 300, 400, 500, 600 MU/min
SSD	100 cm
Applicator^*^	With applicator (10 cm × 10 cm), Without applicator

* This was set manually in the same way as the secondary collimator was set to 20 cm × 20 cm in the CONV mode.

According to the electron beam measurement protocol proposed by IAEA TRS-398, an evaluation was performed under conditions of SSD 100 cm and field size of 10 cm × 10 cm, and the implemented gating control circuit was used to ensure consistent and uniform irradiation. The mean dose rate (*D*_*m*_) was calculated as the ratio of the irradiation time through the gating control circuit and the dose measured on the EBT-XD film, and dose-per-pulse (DPP, denoted as *D*_*p*_) was calculated as the product of *D*_*m*_ and the nominal pulse repetition frequency (PRF, denoted, *f*) of Varian Clinical iX. The instantaneous dose rate (denoted as *D*_i_) was calculated as the ratio of *D*_*P*_ and nominal pulse width (denoted, *w*) of the Varian Clinical iX. The relationships between related physical quantities are summarized by two equations [22]:


$$D_{p}\;\left[\frac{\text{Gy}}{\text{pulse}}\right]=D_{m}\;*\;f$$ (1)

$$D_{i}\;\left[\frac{\text{Gy}}{\text{s}}\right]=D_{p}\;*\;w^{-\text{1}}$$ (2)

In this case, in Varian’s LINAC, *f* is variable in the range from 60 to 360 Hz (i.e., 600 MU/min to 360 Hz, 300 MU/min 180 Hz) according to the dose rate setting in the console, and w is set to 4 μs. In this study, the expected values of *D*_*P*_ and *D*_i_ were determined using nominal values.

### Evaluation of percentage depth dose

**Fig 3:**
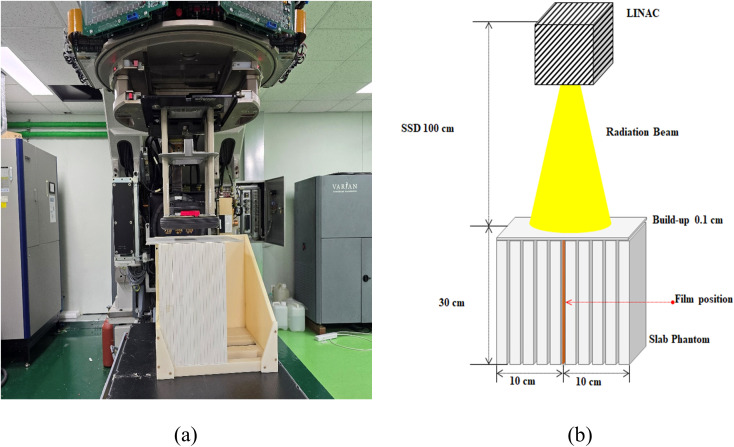
Schematic diagram of the geometrical set-up for the PDD curve experiment. **(a)** Experiment set-up, **(b)** Schematic diagram.

For evaluation of the PDD curve, a 20.3 cm × 25.4 cm EBT-XD film was placed parallel to the central axis in the SP34 slab phantom. For lateral electron equilibrium, the EBT-XD film was placed at the center of the 20 cm side of the SP34 slab phantom and fixed using an Acrylonitrile Butadiene Styrene (ABS) jig (*ρ*_*pl*_ 1.07 g/cm^3^, KIRAMS, Korea). Additionally, to minimize distortion of dose distribution on the surface, a 0.1 cm SP34 slab phantom was placed as a build-up. Fig 3 presents a schematic diagram for a PDD curve evaluation experiment using EBT-XD film.

In this study, the highest dose rate that can be selected in the treatment console was set to 600 MU/min for the evaluation of the PDD curve, and the irradiation time for the evaluation was set to 100 ms. The film reading was performed after 24 hours using the Epson Expression 10000XL to obtain the imaging information in TIFF format, under the same conditions as those for obtaining the calibration curve, as described above. The dose distribution for the red channel was analyzed using DoseLab software. The depth profile was analyzed for the central axis, and the pixel information of the X axis was converted into water equivalent thickness; 72 dpi (≒ 0.03528 cm/pixel), *ρ*_*pl*_ 1.045 g/cm^3^. Through preliminary research, the water-equivalent thickness was calculated based solely on the physical density of the SP34 slab phantom; following international protocol recommendations, no additional correction or scaling factor was applied to the measured depth-dose [43].

Next, for a more quantitative evaluation of the characteristics of the PDD curve, curve fitting was carried out in the units of 0.01 g/cm^2^ using a piecewise cubic hermite interpolating polynomial (PCHIP), and several parameters were analyzed, as follows [44,45]; *z*_*max*_, *z*_*ref*_, *R*_*90*_, *R*_*50*_, *R*_*p*_. *z*_*max*_ is the depth of the maximum dose, *R*_*x*_ is the depth of x percent of the maximum dose. In clinical applications, *R*_*90*_ is defined as the therapeutic range, and *R*_*p*_ is defined as the practical range. In addition, we calculated *z*_*ref*_ and *R*_*p*_ for the evaluation, along with the energy characteristics of the FLASH mode, using the following equations:


$$z_{ref}\;[\text{cm}]=\text{0}.\text{6}\;*\;R_{\text{50}}-\text{0}.\text{1}$$ (3)

$$R_{p}\;[\text{cm}]=\text{1}.\text{27}\;*\;R_{\text{50}}-\text{0}.\text{23}$$ (4)

$$E_{\text{0}}\;[\text{MeV}]=\text{0}.\text{022}\;*\;{\left(R_{\text{50}}\right)}^{\text{2}}+\text{2}.\text{059}\;*\;R_{\text{50}}+\text{0}.\text{656}\;[\text{cm}]$$ (5)

$$E_{p,\text{0}}\;[\text{MeV}]=\text{0}.\text{0025}\;*\;{\left(R_{p}\right)}^{\text{2}}+\text{1}.\text{98}\;*\;R_{p}+\text{0}.\text{22}\;[\text{cm}]$$ (6)

Where, *E*_*0*_ is the mean energy at the phantom surface and *E*_*p,0*_ is the most probable energy at the phantom surface.

### Evaluation of beam profile

**Fig 4:**
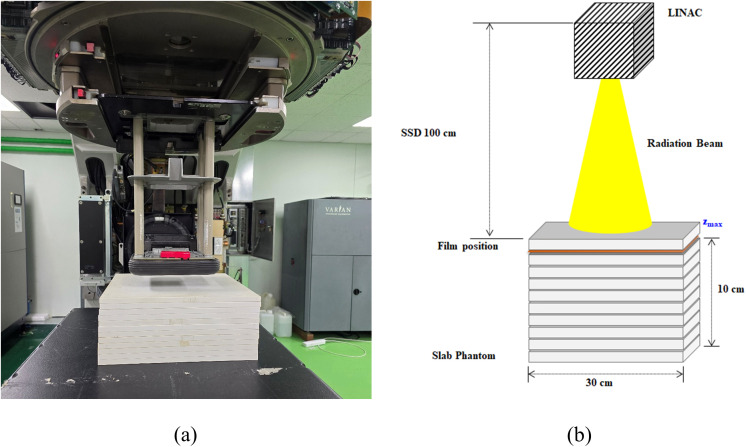
Schematic diagram of the geometrical set-up for the beam profile experiment. **(a)** Experiment set-up, **(b)** Schematic diagram.

To measure the beam profile, EBT-XD film (20.3 cm × 25.4 cm) was placed in the SP34 slab phantom, perpendicular to the electron beam axis. In this case, the EBT-XD film was placed at the *z*_*max*_ position, which was determined in the PDD curve for FLASH mode, and a 10 cm-thick SP34 slab phantom was placed at the bottom of the EBT-XD film to prevent back scattering by the couch. Fig 4 presents a schematic diagram of the experimental setup for the beam profile evaluation.

The beam profile evaluation was performed with a dose rate of 600 MU/min and irradiation time set to 100 ms. For a more quantitative evaluation of the beam profile characteristics, the function of DoseLab software that provides horizontal and vertical lines at the center of the radiation field based on the 50% iso-dose was used. For the acquired beam profile, curve fitting was carried out in the units of 0.01 cm using the PCHIP interpolation method, and several parameters were analyzed as follows; *W*_*95*_, *W*_*90*_, *W*_*50*_. *W*_*x*_ denotes the length of connecting the point of the field at X% of the maximum in the beam profile, which indicates the effective field size. In general, 90% is defined as clinical field size and 50% as a geometrical field size. In this study, 95% was defined as a useful field size for preclinical studies. (i.e., small animal and cell experiments).

Furthermore, the following formulas were used to evaluate the flatness and symmetry along with the effective field size of the FLASH mode [46].


$$\text{Flatness}\;[\%]=\left[\left(D_{max}-D_{min}\right)\cdot {\left(D_{max}+D_{min}\right)}^{-\text{1}}\right]\times \text{100}$$ (7)

$$\text{Symmetry}\;[\%]=\left(D_{max,\;left\;side}\cdot \overset{-\text{1}}{\underset{max,\;\;right\;side}{D}}\right)\times \text{100}$$ (8)

Where, flatness refers to the *D*_*max*_ and *D*_*min*_ in the central 80% of the beam profile. Symmetry is often defined as a maximum permissible percentage deviation of the “left-side” dose from the “right-side” dose of a beam profile, often at 80% of the FWHM points.

### Evaluation of beam output

In this study, the EBT-XD film was used for beam output evaluation. An Advanced Markus Electron Chamber (TM34045, PTW-Freiburg GmbH, Germany) was used to verify output stability. The ionization chamber was not suitable for absolute dosimetry due to the dose rate dependence and saturation phenomenon, but it is useful for relative dosimetry and output stability evaluation [43].

**Fig 5:**
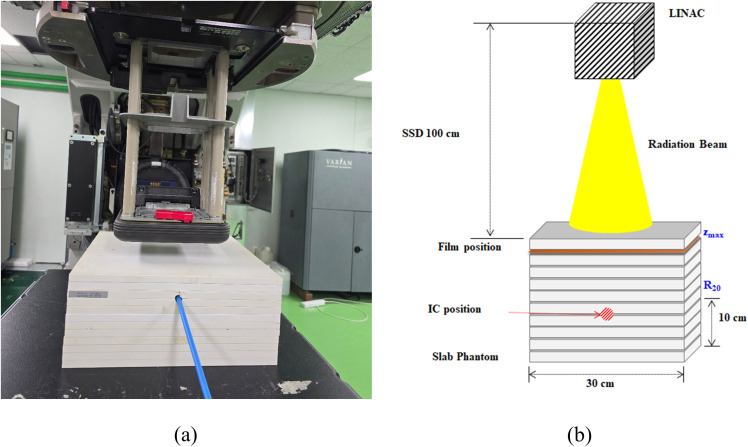
Schematic diagram of the geometrical set-up for the beam output experiment. **(a)** Experiment set-up, **(b)** Schematic diagram.

For measurements of the beam output, EBT-XD film (5 cm × 5 cm) was placed at the *z*_*max*_ position in the SP34 slab phantom, perpendicular to the electron beam axis. The Advanced Markus Electron Chamber was used to verify output stability at the R_20_ position, and a 10 cm-thick SP34 slab phantom was placed at the bottom of the R_20_ position, to prevent backscattering by the couch. Fig 5 presents a schematic diagram of the experimental beam output evaluation setup.

To evaluate the beam output, the irradiation conditions presented in Table 3 were applied with three repeated measurements and a region of interest (ROI) of 2 cm × 2 cm was set from the center of the film, and the mean and standard deviation were calculated. The ROI was set to 2 cm × 2 cm to minimize lateral response artifacts (LRA); for EBT-XD films, LRA within a 30 mm central region is reported to be within 0.5% for doses up to 40 Gy [47]. For the ROI setting, a custom ROI function in DoseLab, which enables direct designation of coordinates in the image, was used to minimize human error; in addition, the central ROI was selected and consistent scanning conditions(films placed at the center of the scanner bed with a fixed orientation) were applied to further minimize potential LRA effects. For more quantitative characterization of the beam output, a linear function for each dose rate was derived by performing a linear regression analysis on the irradiation time based on the calculated results and R-sq (coefficient of determination, denoted R^2^). R-sq generally represents the correlation between the measured signal and the trend of the function, and it is not a direct expression of linearity. However, in this study, the measured signals with respect to the irradiation time were expressed in terms of correlation with a linear function, in the form of “Y = a X + b,” and therefore, R-sq was used as an indirect indicator of linearity. Additionally, *D*_*m*_, defined as the dose per unit time, corresponds to the constant “a” in the linear function of “Y = a X + b.” Finally, through the nominal values of the Varian Clinac iX, parameters that were useful for the output definition were analyzed; *D*_*p*_, *D*_*i*_.

## Results and discussion

### Evaluation of percentage depth dose

**Fig 6:**
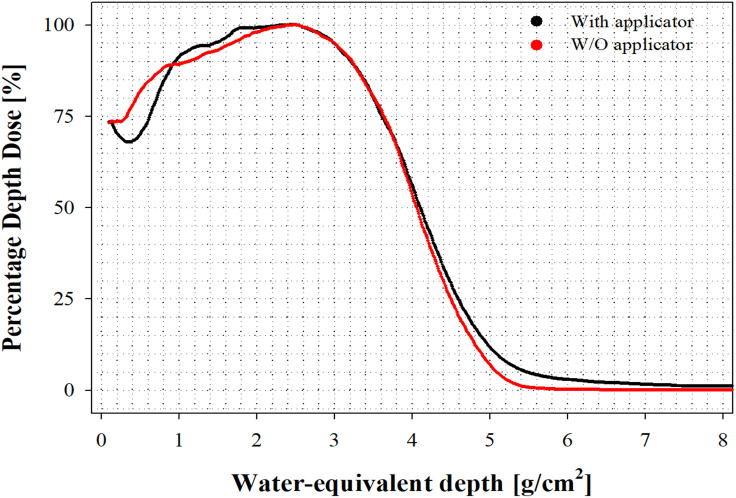
Percentage depth dose as a function of water equivalent depth.

In this study, in order to develop a research platform for FLASH-RT, the FLASH mode was set according to the proposed FLASH modification protocol using a clinical LINAC, and the implemented electron beam quality was characterized. The PDD curve was evaluated with/without an applicator using the EBT-XD film in the SP34 slab phantom. Fig 6 shows the result of the PDD curve of the FLASH electron beam with and without an applicator.

The measured PDD curve showed a similar trend depending on the status of the applicator. Table 4 outlines the beam characterization parameters of the FLASH electron beam.

**Table 4:** PDD parameters for the definition of beam characterization in the FLASH mode.

Applicator	Parameters								
	Range [g/cm^2^]					PDD [%]		Energy [MeV]	
	*z*_*max*_	*z*_*ref*_	*R*_*90*_	*R*_*50*_	*R*_*p*_	1 g/cm^2^	2 g/cm^2^	*E*_*0*_	*E*_*p,0*_
With	2.48	2.55	3.21	4.11	4.99	91.09	99.15	9.49	10.2
Without	2.50	2.51	3.23	4.06	4.93	89.13	97.91	9.38	10.0

As a result of analyzing *R*_*50*_, which is used as a beam quality index for electron beams in clinical practice, the value with an applicator was 4.11 g/cm^2^, and the value without an applicator (denoted, w/o applicator) was 4.06 g/cm^2^. These results indicate that dosimetry using the SP34 slab phantom in clinical LINAC FLASH mode is useful. Additionally, as a result of the *E*_*0*_ and *E*_*p,0*_ calculations, the value with applicator was analyzed as 9.49 MeV (*E*_*p,0*_: 10.2 MeV) and the value w/o applicator was analyzed as 9.38 MeV (*E*_*p,0*_: 10.0 MeV). According to the British Journal of Radiology BJR Supplement 25, the *E*_*0*_ value is presented as 9.91 MeV (*E*_*p,0*_: 10.7 MeV) at 10 MeV (*R*_*50*_: 4.3 g/cm^2^). In the case of CONV mode 9 MeV for the clinical LINAC for research, the E_0_ value was 8.31 MeV (*E*_*p,0*_: 8.82 MeV) [48]. Furthermore, among the pre-clinical FLASH parameters presented by Farr et al. [49], the energy values obtained above satisfied the condition of beam energy ≥ 4.5 MeV.

### Evaluation of beam profile

**Fig 7:**
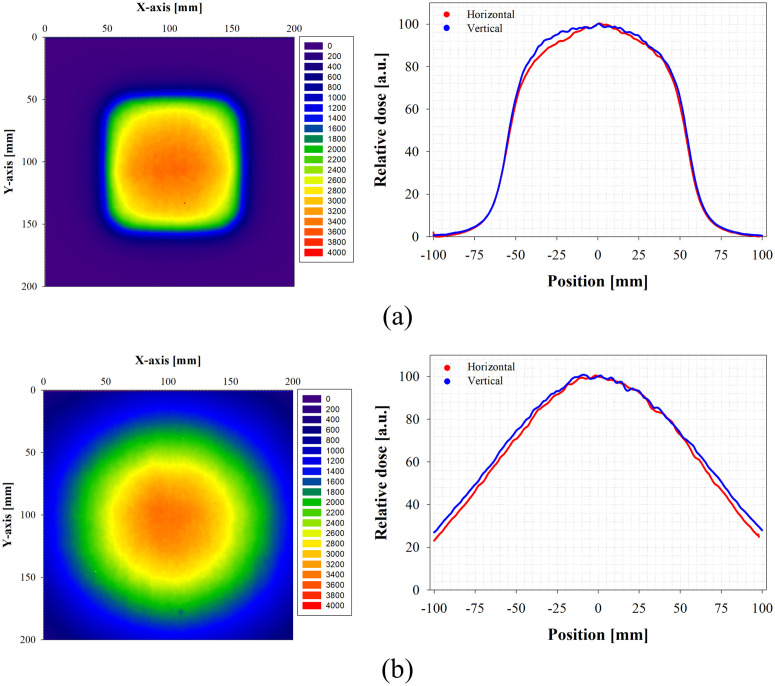
Iso-dose distributions for verification of the beam shape. **(a)** With applicator; iso-dose distribution (left) and beam profile (right), **(b)** Without applicator; iso-dose distribution (left) and beam profile (right).

To evaluate the electron beam geometry of the FLASH mode of the clinical LINAC for research, the beam profile with/without an applicator was measured using the EBT-XD film in the SP34 slab phantom. Fig 7 presents the beam profile results of the FLASH electron beam with/without an applicator.

As a result of a beam profile measurement, in the case with an applicator, the field boundary showed a rectangular shape. However, the internal distribution of the field showed a Gaussian distribution, and the beam profile without an applicator showed a field with an elliptical Gaussian distribution. Table 5 outlines the parameters for the characterization of the effective field size.

**Table 5:** Beam profile parameters for effective field size characterization.

Applicator	Parameters [mm]					
	*W*_*95*_		*W*_*90*_		*W*_*50*_	
	Horizontal	Vertical	Horizontal	Vertical	Horizontal	Vertical
With	33.9	47.0	57.1	65.4	106.3	107.7
Without	37.8	36.6	56.7	58.5	141.7	149.7

As a result of analyzing *W*_*50*_ used as the geometrical field size, the value was 106.3 mm × 107.7 mm in the case with an applicator and ∅ 145.6 mm for the case without an applicator; a diameter of elliptical Gaussian distribution was converted into a circle diameter. The *W*_*90*_ used as a clinical field size was ∅ 61.1 mm with an applicator and ∅ 57.6 mm without an applicator. Also, the *W*_*95*_ was ∅ 39.9 mm with an applicator and ∅ 37.2 mm without an applicator. Our results confirmed that regardless of the presence/absence of the applicator, the beam size parameter condition of (95% iso-dose) ≥ 20 mm was satisfied among the pre-clinical FLASH parameters presented by the Farr et al. [49] Additionally, as a result of the horizontal and vertical line analysis, a dose distribution in the shape of an elongated ellipse was observed in the vertical line direction. It is considered to be because the servo and steering system were turned off in FLASH mode. In particular, the steering information is delivered to the control system from the monitoring chamber placed inside the LINAC, and in the control system, the radial and lateral steering coil currents are adjusted, and the beam steering is controlled by adjusting the intensity of the bending magnet before the beam exits the flight tube [50]. However, since steering information cannot be provided to the control system in FLASH mode, proper adjustment cannot be achieved, and the electron beam shows a wide distribution in the vertical direction.

In addition, beam flatness and symmetry were calculated to analyze the geometry of the electron beam in the FLASH mode. Table 6 presents the geometry characterization parameters of the electron beam in the FLASH mode.

**Table 6:** Parameters for electron beam geometry characterization in the FLASH mode.

Applicator	Parameters [%]			
	Flatness		Symmetry	
	Horizontal	Vertical	Horizontal	Vertical
With	12.7	11.2	99.7	99.8
Without	21.9	21.7	100.5	100.4

As a result of the flatness analysis, with an applicator, the value averaged 11.9%, and without an applicator, the value averaged 21.8%. This is considered to be because we did not place a scattering foil in the beam path in the FLASH modification protocol to implement the FLASH mode (≥ 40 Gy/s). In general, a scattering foil is used for uniform distribution of the electron beam in the field, but Schüler et al. [36] reported through Monte Carlo simulation results that the dose rate could be reduced by more than three times when scattering foils were used. Furthermore, Lempart et al. [37] reported that the dose rate could be reduced by more than 2.68 times. Therefore, in order to meet the ± 1% tolerance proposed in the American Association of Physicists in Medicine (AAPM) TG-142 while maintaining a dose rate appropriate for FLASH mode, further multifaceted research (i.e., scattering foil with manual tuning, applicator, etc.) is necessary [51]. As a result of the symmetry analysis, the average value with an applicator was −0.25%, and that without an applicator was 0.45%. This satisfies the tolerance of ± 1% proposed in AAPM TG-142.

### Evaluation of beam output

**Fig 8:**
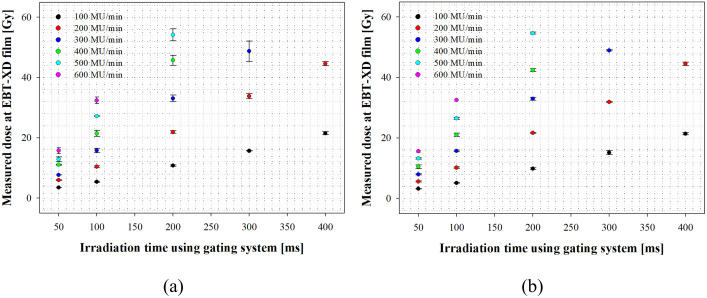
Measured dose as a function of irradiation time. **(a)** With applicator, **(b)** Without applicator.

In this study, in order to evaluate the dosimetric characteristics of electron beams of the FLASH mode, the output with/without an applicator was measured according to the conditions presented in Table 3 using the EBT-XD film in the SP34 slab phantom. Fig 8 shows the output measurements of the FLASH electron beam with/without an applicator.

Table 7 shows the parameters related to the output characteristics measured through the EBT-XD film in the FLASH mode. In this case, in the linearity function, Y denotes the output measured using the EBT-XD film, and X indicates the time the electron beam is irradiated through the gating system; Since R-Sq ≥ 0.99, the linearity in the relationship is confirmed.

**Table 7:** Output values measured through EBT-XD film in FLASH mode.

Applicator	Setting Dose-rate	Linearity function	R-Sq	Parameter		
				*D*_*m*_ [Gy/s]	*D*_p_* [Gy/pulse]	*D*_*i*_ [Gy/s]
With	100	Y = 0.0517X + 0.5029	0.9976	51.7	0.862	2.15 × 10^5^
	200	Y = 0.1122X – 0.2382	0.9991	112.2	0.935	2.34 × 10^5^
	300	Y = 0.1652X – 0.5280	0.9995	165.2	0.918	2.29 × 10^5^
	400	Y = 0.2328X – 1.1046	0.9987	232.8	0.970	2.43 × 10^5^
	500	Y = 0.2745X – 0.5971	0.9998	274.5	0.915	2.29 × 10^5^
	600	Y = 0.3330X – 0.8990	1.0000	333.0	0.925	2.31 × 10^5^
Without	100	Y = 0.0519X + 0.0286	0.9945	51.9	0.865	2.16 × 10^5^
	200	Y = 0.1111X – 0.5747	0.9984	111.1	0.926	2.31 × 10^5^
	300	Y = 0.1651X – 0.4339	0.9997	165.1	0.917	2.29 × 10^5^
	400	Y = 0.2133X – 0.2320	1.0000	213.3	0.889	2.22 × 10^5^
	500	Y = 0.2772X – 0.8823	0.9998	277.2	0.924	2.31 × 10^5^
	600	Y = 0.3391X – 1.3754	1.0000	339.1	0.942	2.35 × 10^5^

* Pulse values were estimated based on the minimal pulse repetition frequency(PRF).

The beam output measurements showed that regardless of the presence/absence of an applicator, the beam output exceeded the dose limit of EBT-XD (≤ 60 Gy) under the condition of 600 MU/min and ≥ 200 ms irradiation time, 400 and 500 MU/min and ≥ 300 ms irradiation time and 300 MU/min and ≥ 400 ms irradiation time; we excluded measurement conditions corresponding to the over-range of EBT-XD from the analysis.

As a result of the *D*_*m*_ measurements, most of the dose rate settings that were controllable from the treatment console fell under the FLASH beam condition (≥ 40 Gy/s), with the exception of the 100 MU/min condition (*D*_*m*_: 51.7 Gy/s with applicator, *D*_*m*_: 51.9 Gy/s without an applicator). Our research confirmed that regardless of the presence/absence of an applicator, the *D*_*m*_ results satisfied the condition of average dose rate ≥ 40 Gy/s, among the pre-clinical FLASH parameters presented by Farr et al. [49]. Additionally, we analyzed the beam output linearity according to the controllable dose rate condition in the treatment console. As a result, with an applicator, a trend with a linear function “Y = 0.5603 X – 1.2” was observed, with R-Sq = 0.9975, and without an applicator, a trend with a linear function of “Y = 0.5664 X – 5.3” was observed, with R-Sq = 0.9983.

Also, D_p_ and D_i_ were calculated using the nominal values of the clinical LINAC. As a result of the *D*_*p*_ analysis, it was 0.915–0.970 Gy/pulse with an applicator and 0.889–0.942 Gy/pulse without an applicator; the *D*_*p*_ for 100 MU/min, which does not correspond to the FLASH beam condition, was excluded. The results did not satisfy the pulse dose values (dose per macro pulse) 1 Gy/pulse among the pre-clinical FLASH parameter proposed by Farr et al. [49]. However, it should be noted that in this study, it did not reflected a major issue that considered in the FLASH mode; instability of the generated initial pulse after beam-on (i.e., ramp-up time). According to Ashraf et al. [24] ramp-up is a phenomenon caused by the delay (≤ 36 ms) of the automatic frequency control (AFC) after beam irradiation, and the dose of the initial 4–5 pulses has been reported to be about 30%. Therefore, many of the FLASH research groups are investing substantial resources and time in developing a beam pulse monitoring (BPM) system that enables real-time measurement of beams independently of the gating system for electron beam irradiation. Considering the impact of the ramp-up phenomenon through the establishment of the BPM system in the future, it is thought that dose rate ≥ 1 Gy/pulse can be expected from the clinical LINAC. As a result of the *D*_*i*_ analysis, the value was 2.29 × 10^5^–2.43 × 10^5^ Gy/s with an applicator and 2.16 × 10^5^–2.35 × 10^5^ Gy/s without an applicator.

## Conclusion

In this study, to develop a research platform for FLASH-RT, the FLASH mode was set according to the proposed FLASH modification protocol using the clinical LINAC for research installed in Korea Institute of Radiological & Medical Sciences. The implemented FLASH electron beam characteristics (i.e., PDD, beam profile, output) were evaluated using the EBT-XD film.

As a result of the measurement of the FLASH mode beam characteristics, the energy of the beam irradiated from the clinical LINAC has a nominal 9–10 MeV beam quality, which is the quality used in clinical practice; 9.49 MeV (*E*_*p,0*_: 10.2 MeV). The results of this study are expected to be utilized to determine the measurement depth in routine tests (i.e., output constancy, energy constancy, flatness, & symmetry constancy) for the clinical LINAC for research and are expected to be utilized as basic data in various preclinical studies in the future. Furthermore, at the *z*_*max*_ position, the field size was 106.3 mm × 107.7 mm, with a Gaussian dose distribution. The value of symmetry averages −0.25%, which is within the tolerance (± 1%), but the flatness averages 11.9%, which deviates from the tolerance (± 1%). The effective field sizes were *W*_*95*_ ∅ 39.9 mm and *W*_*90*_ ∅ 61.1 mm respectively. Finally, regarding the output characteristics, except for the condition of 100 MU/min among the dose rate setting conditions that can be set in the treatment console, the overall output values fell under the FLASH beam condition (≥ 40 Gy/s). Furthermore, the output showed linearity for the dose rate setting conditions; Max. *D*_*m*_ 333.0 Gy/s, *D*_*p*_ 0.915–0.970 Gy/pulse, *D*_*i*_ 2.29 × 10^5^–2.43 × 10^5^ Gy/s. Based on the results of this study, the values of the pre-clinical FLASH parameters presented by Farr et al. [49] were mostly satisfied. In theory, considering the “ramp up” phenomenon, the pulse dose is expected to be ≥ 1 Gy/pulse, but since it was not determined experimentally in this study, additional research will be conducted. In addition, in Fig 8, some variability in dose rate over time was observed. This variation is primarily due to the use of the application (electron cone) and statistical accumulation of dose measurements, rather than intrinsic instability of the accelerator. The application modifies the beam shaping and scattering mechanisms, making the measured dose more sensitive to small shot-to-shot differences. While these differences are minimal for short irradiation times, they accumulate over longer periods, leading to increased standard deviation. Therefore, the observed fluctuations mainly reflect the influence of the application and statistical effects, rather than time-dependent degradation of the accelerator.

We plan to build a research platform so that the FLASH mode implemented through the clinical LINAC for research can be used for preclinical studies, to elucidate the FLASH mechanism, and for a wide range of research and industrial field applications that require FLASH beam conditions. Furthermore, this study also reaffirmed the necessity for two major issues required for the development of a FLASH research platform; (i) Research on flatness improvement without compromising the dose rate corresponding to the FLASH beam condition, and (ii) Development of a BPM system that considers the “ramp-up” time. For future research, we plan to investigate the use of scattering foil with manual tuning, develop a novel applicator to improve flatness, and research different types of radiation detectors (i.e., solid-sate radiation detector, plastic scintillation detector, etc.) for BPM system development.
